# Revisiting the streptococcal M protein: modern perspectives on antibody function, host interactions, and therapeutic targeting

**DOI:** 10.3389/fimmu.2026.1771053

**Published:** 2026-02-03

**Authors:** Sebastian Wrighton, Pontus Nordenfelt

**Affiliations:** 1 Department of Clinical Sciences Lund, Division of Infection Medicine, Faculty of Medicine, Lund University, Lund, Sweden; 2 Institute of Immunology, Center for Pathophysiology, Infectiology and Immunology, Medical University of Vienna, Vienna, Austria; 3 Department of Laboratory Medicine, Clinical Microbiology, Skåne University Hospital, Lund University, Lund, Sweden; 4 Science for Life Laboratory, Department of Clinical Sciences Lund, Lund, Sweden

**Keywords:** adjunctive therapies, antibody function, group A *Streptococcus*, host interactions, M protein, monoclonal antibodies, phagocytosis, *Streptococcus pyogenes*

## Abstract

Group A *Streptococcus* (GAS) causes extensive global morbidity and rising rates of invasive disease, for which clinical outcomes remain poor despite antibiotic treatment of susceptible strains. This limitation of current therapy underscores the need for alternative or adjunctive approaches. Antibody-based interventions represent a promising but underexplored strategy. Historically, the streptococcal M protein was considered an unsuitable therapeutic target due to its sequence variability and concerns about autoreactivity. These perceptions arose largely from early murine hybridoma studies, peptide-based immunizations, and functional assays that disproportionately emphasized hypervariable epitopes. Recent advances now challenge these longstanding views. Improved structural and functional analyses, human monoclonal antibody discovery, and more sensitive measurements of phagocytosis and opsonization demonstrate that conserved regions of M protein can support effective immune engagement. Newly described mechanisms, including dual-Fab binding and antibody-dependent remodeling of the bacterial surface, further reveal unexpected layers of antibody function during GAS infection. This review integrates historical and modern insights into M-protein immunobiology and discusses how antibody engineering may enhance therapeutic activity. We also consider how monoclonal antibodies could be deployed alongside antibiotics and adjunctive treatments. Together, these developments support a reassessment of M protein as a viable target for antibody-based therapies against GAS.

## Group A *Streptococcus* – a highly evolved human pathogen

### Current challenges and the need for novel therapeutics

The human immune system has co-evolved with Group A *Streptococcus* (GAS), or *Streptococcus pyogenes*, leading to its remarkable ability to adapt and persist within human populations (1). Current estimates suggest that around 20 percent of children are asymptomatic carriers of GAS, with markedly lower rates among adults (2). While GAS typically evades our immune systems effectively, it can also cause a broad range of diseases. These include mild superficial infections like pharyngitis and impetigo. Although these infections are generally self-limiting, they should be treated with antibiotics since repeated GAS infections substantially increase the risk of developing post-streptococcal inflammatory syndromes such as rheumatic heart disease (RHD). Additionally, GAS can more rarely cause severe invasive diseases, including streptococcal toxic shock syndrome (STSS) and necrotizing fasciitis (3).

GAS causes approximately 700 million symptomatic infections annually (4). While these are mainly superficial, estimates suggest that GAS infections result in over 500,000 deaths per year, primarily caused by RHD and invasive disease manifestations. The overwhelming burden of GAS disease falls on low-income regions (the Global South) and marginalized groups even in high-income countries, where limited access to healthcare prevents prompt antibiotic treatment (5). However, even in these countries, reports have shown a steady rise in deadly GAS infections as well as postinfection autoimmune sequelae (6). A particularly dramatic example occurred after the social distancing measures put in place due to the COVID-19 pandemic were lifted. A sharp rise in severe invasive GAS infections was reported globally, especially amongst children (7).

Notably, GAS treatment has changed little since the discovery of antibiotics. Unlike many other bacterial pathogens, GAS has retained its sensitivity to penicillin and its derivatives. However, in recent years, this trend has begun to shift with increased reports of GAS strains showing reduced β-lactam sensitivity (8). Additionally, US treatment guidelines recommend combining β-lactams with clindamycin for greater effectiveness in severe GAS infections (9), but over the past decade, resistance to macrolides and clindamycin has risen sharply (from 13% to 33%) (10). This suggests that soon, even in the Global North, treating what were once simple superficial infections may become challenging. Such a development could lead to a significantly higher incidence of poststreptococcal autoimmune complications worldwide, along with increasingly difficult-to-treat invasive infections. Therefore, it is crucial to prioritize the development of novel and effective preventive and therapeutic strategies.

### Diversity of M protein serotypes: clinical and pathogenic implications

A defining feature of *Streptococcus pyogenes* biology is the extraordinary diversity of its M protein, encoded by the *emm* gene (11). Sequence variation within the N-terminal hypervariable region has led to the classification of more than 275 distinct *emm* types (12), often referred to as M serotypes. This diversity is not merely taxonomic; it has profound consequences for immune recognition, epidemiology, and clinical disease manifestations, and it represents one of the central obstacles to both vaccine- and antibody-based therapeutic development.

Clinically, different M protein serotypes are strongly associated with specific disease phenotypes and tissue tropisms. Certain *emm* types, such as *emm1*, *emm3*, *emm12*, and *emm28*, are disproportionately represented among invasive GAS infections, including necrotizing fasciitis and STSS (13). Other serotypes are more commonly linked to superficial infections such as pharyngitis or impetigo (14). However, it is important to note that because invasive disease manifestations have extremely high mortality rates, they represent an evolutionary dead end for the infectious agent, as transmission to other hosts is far less likely. Moreover, although invasive disease has been associated with certain *emm* types, it is more closely linked to individual host risk factors (15, 16). Overall, these associations between certain *emm* types and specific disease outcomes are not absolute but reflect differences in virulence factor expression, regulation, and host interactions that are at least partly mediated by the M protein itself. For example, highly invasive *emm1* strains often express M proteins with strong fibrinogen-binding capacity, promoting immune evasion and excessive inflammation in the bloodstream (17).

M protein diversity also underpins marked differences in pathogenic mechanisms among serotypes. Structurally, M proteins can be grouped into patterns (A–C, D, and E), based on the presence or absence of specific repeat regions (18). For example, A–C pattern M proteins are frequently associated with throat infections and invasive disease (13, 15) and often possess extended, unstable N-terminal regions that can sequester a wide range of host factors, thereby contributing to immune evasion (19–22). These structural differences influence how each serotype recruits host proteins such as fibrinogen, complement regulators, or immunoglobulins, thereby shaping the local immune landscape and determining the bacterium’s capacity to resist opsonization (23, 24). From an immunological perspective, serotype diversity enables GAS to persist at the population level through immune escape. Antibodies elicited against the hypervariable region of one M type typically confer little or no protection against others, allowing reinfection with heterologous strains throughout life (25). This phenomenon explains both the high prevalence of repeated GAS infections and the historical focus on type-specific immunity. However, it also creates a paradox: while type-specific antibodies can be strongly opsonic, they offer narrow protection, whereas antibodies targeting conserved M protein regions may provide broader coverage but vary widely in functional efficacy (26–28).

Importantly, not all serotypes are equivalent in their interactions with antibodies. Differences in M protein length, conformational dynamics, and host-protein binding can modulate antibody accessibility and downstream effector functions. As a recent study demonstrated, monoclonal antibodies can show markedly different opsonic activity across *emm* types (26), underscoring that pathogenicity is an emergent property of both serotype and host context. Together, the extensive serotypic diversity of the M protein explains much of GAS’s epidemiological success and clinical heterogeneity. Any effective therapeutic strategy – particularly those centered on M protein targeting – must therefore account not only for antigenic variation, but also for the functional and pathogenic differences that distinguish one M protein serotype from another.

### Importance of vaccine development and adjunctive therapies

As is often the case from a public health perspective, preventing disease should always be the main goal. This approach is also most beneficial economically, as vaccines require infrequent administration and can reduce the need for future medical treatments. This is especially critical for the Global South, where many lack the financial resources to afford medication for repeated superficial GAS infections. Therefore, it is not surprising that the pursuit of a GAS vaccine began over 100 years ago (29). However, these efforts ended quickly in 1969 when a vaccine triggered cases of acute rheumatic fever. Vaccine research resumed after the ban was lifted in 2006 and has advanced toward candidates based on different GAS components (30). Even the most effective vaccines can never fully prevent disease - particularly if large portions of the population remain unvaccinated. Additionally, some individuals cannot be vaccinated or are at risk because they are severely immunocompromised. This means that in the event of an antibiotic-resistant GAS infection, new treatments will be necessary.

As previously mentioned, current therapy for GAS infections involves eradicating the bacteria with antibiotics, particularly the β-lactam antibiotic penicillin (31). Cephalosporins, clindamycin, and macrolides can be administered in the case of allergy to penicillin (32–34). For severe, invasive infections, studies have found that a combination of a β-lactam and clindamycin can effectively reduce mortality (35, 36). Additional treatments such as corticosteroids and vasopressors are often used, but these only stabilize the patient and do not eliminate the infection source (37). Surgery is unfortunately not uncommon when antibiotics are administered too late and bacterial infection has already led to widespread soft tissue necrosis in conditions such as necrotizing fasciitis and myositis (38). This real lack of curative therapies often leaves physicians searching for anything that might help their critically ill patients. Therefore, high-dose pooled intravenous immunoglobulins (IVIG) are sometimes administered. The rationale is that a portion of the antibodies from many healthy donors will recognize GAS antigens, due to the widespread exposure of GAS to the general population (39, 40). These antibodies may work by neutralizing GAS virulence factors or by marking the bacteria so they can be phagocytosed by certain leukocytes, a process called opsonization, first described by Metchnikoff for GAS in 1887 (29). While the idea of using IVIG is reasonable, the reality is more complicated. Many studies have examined the efficacy of IVIG in treating severe infections. Results so far have been inconclusive: some show benefit, others show little to no effect (41, 42). A significant challenge is the lack of standardization in IVIG preparations – since they are human-derived biologics, batches vary in their content of GAS-specific antibodies, leading to inconsistent results, for instance in the ability to neutralize the bacterial enzyme SpeB (43). Additionally, such therapies carry risks like aseptic meningitis, renal dysfunction, and hemolysis (44). Moreover, as with all human-derived biologics, there is a risk of transmitting bloodborne diseases if proper screening isn’t followed (45). We believe these issues could be addressed by developing highly specific therapeutic monoclonal antibodies (mAbs) against GAS.

### A complex and dynamic surface landscape driven by M protein

**Figure 1:**
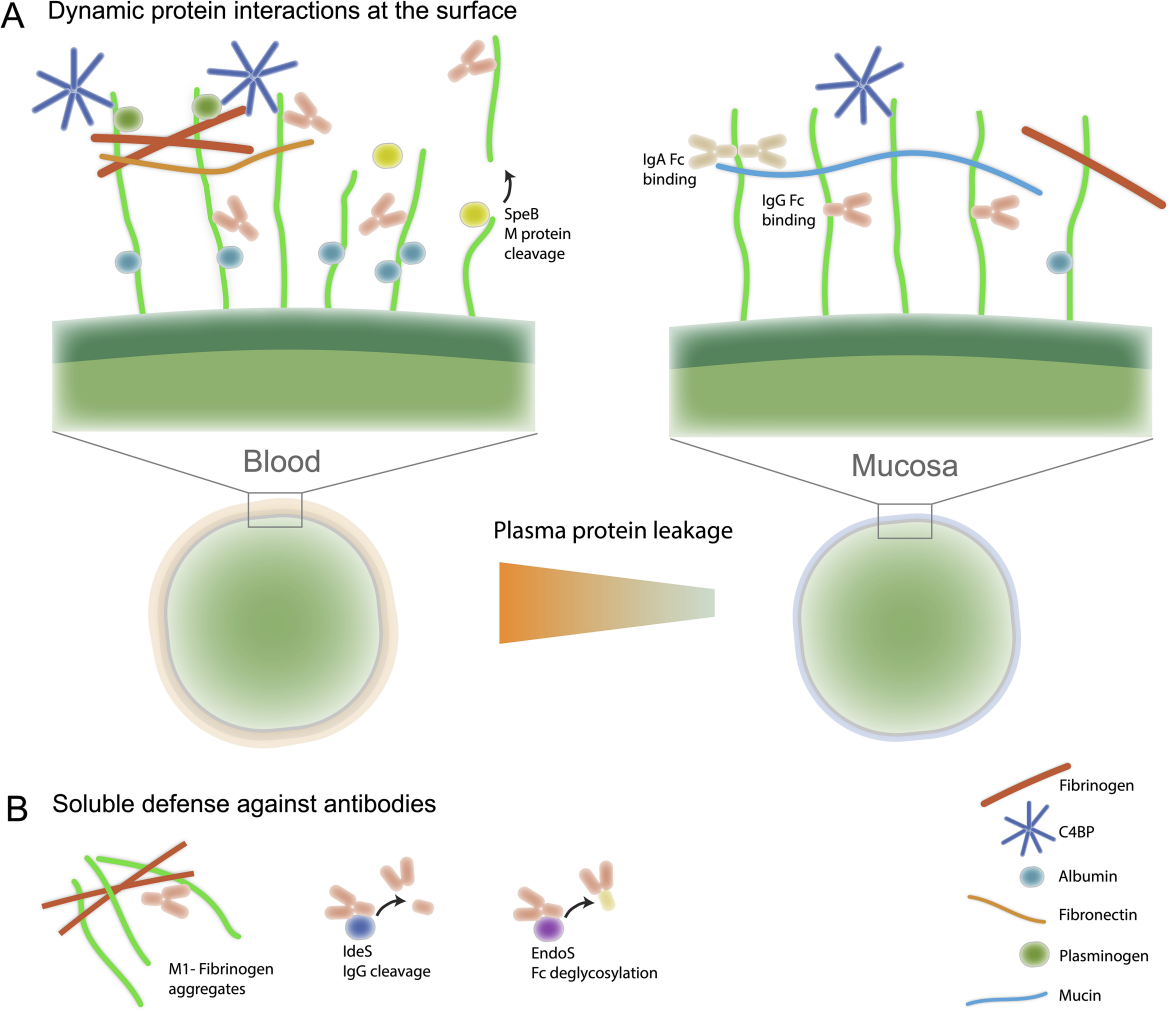
The complex and dynamically remodeled surface landscape of GAS. **(A)** GAS secretes a range of virulence factors that interfere with antibody function. These include shed or cleaved M protein that diverts antibodies from intact bacteria, the IgG-specific protease IdeS that cleaves antibodies at the hinge, and the endoglycosidase EndoS that removes Fc glycans and impairs Fc-mediated effector functions. Together, these secreted factors reduce the effectiveness of naturally acquired or therapeutic antibodies. **(B)** The GAS surface is continuously remodeled through M-protein-mediated recruitment of host proteins. Depending on environmental context and *emm* type, M protein binds distinct sets of plasma or mucosal components—including fibrinogen, albumin, fibronectin, plasminogen, factor H, C4b-binding protein, mucins, and immunoglobulins—resulting in a dynamic “host-protein cloak.” This layering modulates opsonization efficiency, alters antibody accessibility to bacterial epitopes, and drives extensive variation in the observed surface proteome across niches.

Antibodies that reach GAS encounter a highly dynamic and environmentally responsive surface. Beyond the array of native bacterial surface proteins and the prominent hyaluronic acid capsule, which provides a non-immunogenic shield against phagocytosis, GAS recruits numerous human plasma or mucosal proteins to its surface. Central to this remodeling is the M protein, a fibrillar coiled-coil molecule that serves as a multipurpose scaffold for binding diverse host proteins—including fibrinogen, albumin, fibronectin, plasminogen, factor H, and C4b-binding protein (46) (**Figure 1A**). These interactions are both *emm*-type and context-dependent: in the bloodstream, M protein predominantly binds fibrinogen and complement regulators, forming a cloak that inhibits opsonization and phagocytosis; in mucosal environments like saliva, M protein interacts with different sets of host proteins such as mucins or salivary immunoglobulins (20). This dynamic layering of host proteins is not merely decorative; it actively reshapes how the immune system perceives the bacterium and can sterically block antibody access to conserved epitopes (47). Additionally, proteomic studies show that the GAS surface proteome and the layer of bound host proteins vary significantly across environments, highlighting the adaptability of M protein-host interactions (48, 49). Understanding this adaptive surface remodeling is vital for designing mAbs that remain effective across multiple infection sites.

### Soluble M protein pathology and potential antibody benefits

Beyond its surface role, M protein can be shed in soluble form, which directly contributes to pathology (**Figure 1B**). Free M protein forms complexes with human fibrinogen, triggering massive neutrophil activation and vascular leakage, thereby underpinning the systemic toxicity of invasive GAS infections (50). Thus, a monoclonal antibody targeting M protein might neutralize these soluble complexes and mitigate the hyperinflammatory cascade. While, to date, no studies have directly demonstrated that anti-M antibodies can neutralize soluble M protein–fibrinogen complexes *in vivo*, this remains an attractive hypothesis. Indeed, subsequent work showed that certain anti-M1 antibodies can modulate or even potentiate this response under specific conditions (51, 52), highlighting both the therapeutic promise and the mechanistic complexity of targeting M protein. This pathological role of soluble M protein highlights why simply measuring antibody binding is insufficient; functional assays such as neutralization, phagocytosis, and complement activation are essential.

### GAS employs highly sophisticated strategies to evade antibody-mediated immunity

GAS has evolved an exceptional arsenal to neutralize or evade human antibodies (**Figure 1B**). M protein can not only bind the Fc region of IgG and IgA (53, 54), thereby inverting antibody orientation and preventing Fc receptor engagement, but also serves as a scaffold for recruiting host immune regulators such as C4b-binding protein (C4BP) and factor H (55, 56), which suppress complement activation on the bacterial surface, diminishing synergy with antibody opsonization. Most critically, GAS secretes specialized enzymes that directly disable antibodies: IdeS cleaves IgG at the hinge region, leaving F(ab)’2 and Fc fragments, blocking opsonization (57); EndoS removes conserved N-glycans from the Fc domain of IgG, impairing Fcγ receptor binding and downstream effector functions (58). These immune evasion strategies are context-dependent: expression of IgG-modifying enzymes and Fc-binding domains varies across strains and is modulated by environmental cues, such as the shift from mucosal to systemic infection. Compounding this, GAS can mask its surface with host proteins such as albumin, fibronectin, and fibrinogen, potentially forming a barrier that impedes access by specific anti-M antibodies (54, 59, 60). Together, these redundant and dynamic systems make GAS remarkably resistant to antibody-mediated clearance, posing significant challenges for the development of vaccines and therapeutic antibodies.

## Targeting the M protein: opportunities and challenges

### Early studies of anti-GAS mAbs and autoimmunity

The therapeutic potential of antibodies against bacterial infections has deep historical roots. Passive immunization with polyclonal sera, pioneered by von Behring and Kitasato in the late 19th century, first demonstrated that circulating antibodies could neutralize microbial toxins (61). Although human polyclonal preparations remain in limited clinical use (62), their heterogeneity and variable specificity motivated the shift toward monoclonal antibody (mAb) technologies, which offer greater reproducibility and defined antigen targeting.

The development of hybridoma technology in the 1980s enabled the production of murine mAbs against various GAS antigens, and many of these studies focused on the relationship between GAS infection and autoimmunity. Murine mAbs raised against GAS surface proteins, including the M protein and the group A carbohydrate, revealed that some clones cross-reacted with cardiac and cytoskeletal proteins such as myosin, keratin, and actin (63–66). These findings supported the hypothesis that molecular mimicry drives autoimmune pathology in rheumatic heart disease (67) and helped identify N-acetyl-β-D-glucosamine and epitopes within M protein as major autoreactive determinants (68, 69). Early human mAbs derived from tonsillar or peripheral lymphocytes of previously infected individuals also exhibited autoreactivity, with some clones binding both myosin and recombinant M6 despite being raised against an M5 strain (64). These pioneering efforts clarified how GAS infection can generate cross-reactive antibodies, but they provided limited insight into which antibody specificities were actually protective.

A crucial limitation of many early mAb studies was the absence of assays that directly measured immunological function. Investigators often catalogued binding and cross-reactivity, but few assessed whether these mAbs could opsonize or facilitate killing of GAS. An early systematic attempt to evaluate functionality came in 1988, with a test of 19 murine mAbs against native M6 protein for bactericidal activity in human blood. Only one clone, targeting the N-terminal hypervariable region (HVR), enabled complete killing (70). Subsequent peptide immunizations reinforced this finding, establishing the widespread assumption that only type-specific HVR antibodies are protective—an idea that shaped M-protein vaccine development for decades (30, 71).

However, several methodological and structural factors undercut the generality of these conclusions. First, murine antibodies interact inefficiently with most human Fc receptors (72, 73), raising the possibility that many “non-functional” murine mAbs would have behaved differently if expressed as human IgG subclasses (and the ones used were not known). Second, the bactericidal assay used in these classical studies measures only endpoint survival and cannot distinguish failure of opsonization from bacterial resistance to downstream killing mechanisms. Third, the immunogens used in these experiments were often short peptides from structurally unstable regions of M protein.

**Figure 2:**
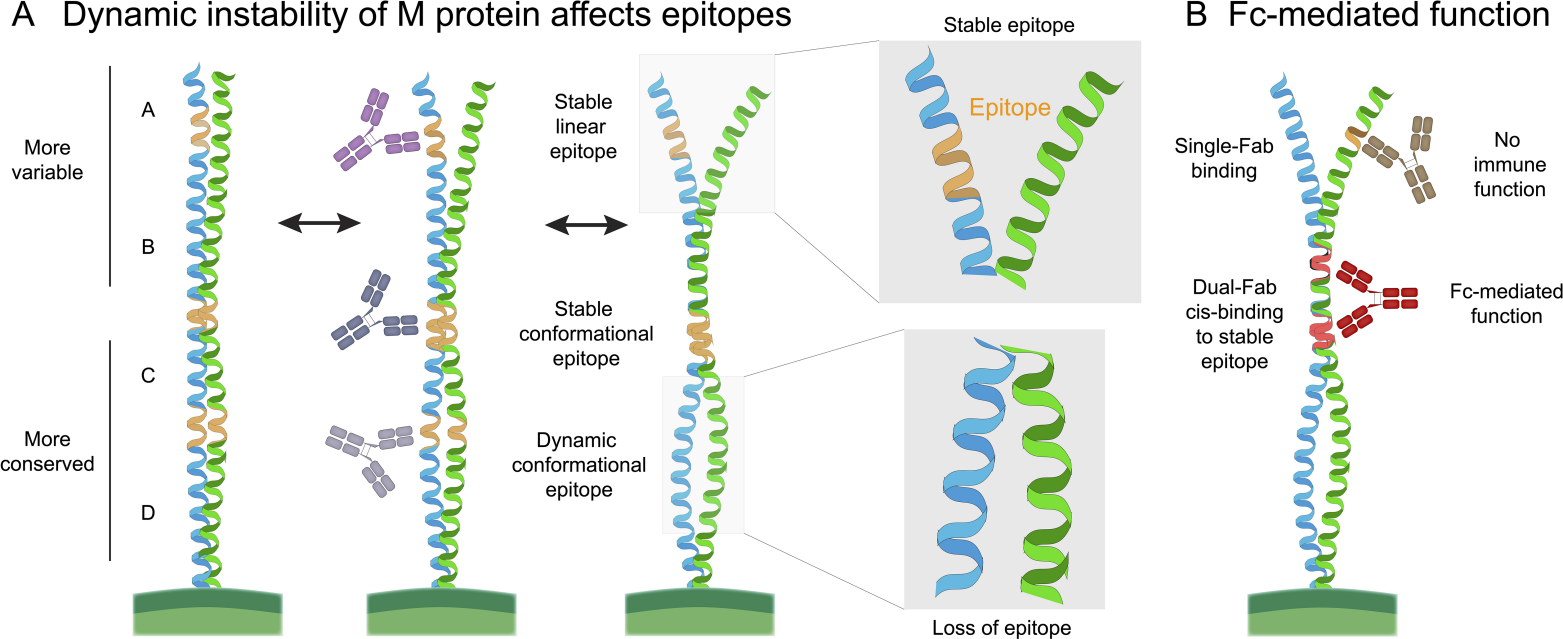
Structural dynamics of streptococcal M protein and implications for antibody targeting. **(A)** M proteins are coiled-coil dimers composed of characteristic sequence motifs called repeats. All M proteins contain C and D repeats, whereas only a subset also contains A and B repeats in the N-terminal region. These upper repeats are enriched in destabilizing residues that promote local unfolding and refolding of the coiled-coil, creating substantial conformational flexibility. This dynamic behavior, combined with the dimeric nature of the molecule, generates a mixture of linear and conformational epitopes that may shift between stable and transient states, complicating epitope targeting and antibody design. **(B)** The M protein comprises both highly variable and conserved regions. Although antibodies can bind across the length of the molecule, only a small subset of epitopes elicit functional, opsonic responses. Recent work identified a human monoclonal antibody capable of a distinctive “dual-Fab cis” binding mode, where both Fab arms simultaneously engage two different epitopes on the same M protein. This unusual geometry stabilizes binding and may provide a promising blueprint for therapeutic antibody development.

M proteins, including the prototypical M6 (an A–C type M protein), contain A/B repeat regions enriched in heptad-disrupting residues that destabilize the coiled coil and generate a spectrum of conformations (19, 74, 75) (**Figure 2A**). In contrast, the C-terminal repeats form stable, rigid coiled coils. Short peptides from the inherently unstable HVR more closely resemble their native structural ensemble than peptides derived from the more stable conserved regions, which require the full coiled-coil context to fold correctly. As a result, peptide immunization in mice was structurally biased toward producing protective HVR-specific antibodies, not because deeper conserved epitopes are non-functional, but because they were not presented in a native-like conformation. This raises the important question of which protective antibodies may have been overlooked due to the use of improperly folded peptide immunogens. Future immunization strategies that stabilize M-protein segments on an ideal coiled-coil scaffold (76) could help reveal a broader functional epitope landscape.

By the 2010s, improved B-cell screening methods and more sensitive functional assays enabled the identification of human mAbs with diverse activities against GAS. These included murine and human mAbs targeting FbaA or SsE with varying degrees of prophylactic and therapeutic benefit (77, 78). Taken together, the historical record highlights both the conceptual progress made and the limitations imposed by early methodologies. Many long-standing assumptions, especially the idea that only HVR-specific antibodies are functional, reflect the combined effects of species-mismatched Fc interactions, insensitive bactericidal assays, and the structural peculiarities of M-protein peptide immunogens. Correspondingly, newer antigenomics-based serology has shown that conserved streptococcal proteins elicit robust IgG in humans (40), suggesting that earlier peptide-focused immunization strategies underestimated the range of naturally targeted epitopes. Updating these frameworks is essential for modern therapeutic antibody development.

### Human mAbs targeting M protein

Concurrently, advances in antibody discovery, such as single B-cell sorting and high-throughput sequencing, enabled isolation of human mAbs against GAS M protein. In a recent study we identified several human mAbs binding the conserved central region of M protein. One antibody, designated Ab25, exhibited a particularly notable binding mechanism, with both Fab arms simultaneously recognizing distinct epitopes on the same M protein molecule – representing a novel ‘dual-Fab cis binding’ mode) (26)(**Figure 2B**). This unique binding mode may explain why, when multiple antibodies target particular regions of M protein, those that exert dual-Fab cis binding can trigger a stronger immune response. The specific epitope is likely critical as well. Importantly, Ab25 not only triggered phagocytosis and NF-κB signaling, it also recognized a conserved epitope pair spanning the B-C domain, allowing it to bind and opsonize a wide range of *emm* types (26).

Similarly, polyclonal IgG in plasma from patients recovering from invasive GAS infection was found to contain opsonic, cross-reactive antibodies that recognized multiple *emm* types. However, somewhat surprisingly, the patient’s own convalescent serum antibodies were, to a greater extent, non-opsonic against that patient’s infecting *emm* type (79). In other words, the broad polyclonal response in plasma had functional gaps for the specific strain involved. Despite this complexity, these findings suggest that cross-reactive Abs may confer enhanced protection both through their ability to recognize multiple strains and their intrinsically improved functional properties. These and similar findings (80) counter previous assumptions that protective, bactericidal antibodies must be *emm* type specific and will likely bolster efforts to develop a vaccine based on a conserved segment of the M protein since this could be a straightforward approach to induce a narrow epitope assembly, yielding a more focused immune response capable of protecting against a broad range of *emm* type strains (81).

### Complex mAb interactions with GAS

Our work highlights that antibody binding can, in some cases, influence the molecular composition of the GAS surface. We observed that engagement of the M protein by certain mAbs increased fibronectin deposition on the bacterial surface, an effect observed not only with the strongly opsonic Ab25 but also with the non-opsonic Ab49 antibody (22). This suggests that antibody-induced modulation of host-factor binding may be a broader property of M-protein–specific antibodies rather than a feature unique to any single clone. Importantly, this enhancement occurred mainly under conditions of low competing antibody levels, such as saliva or low-serum environments, and was reduced or absent when physiological concentrations of polyclonal IgG were present, indicating that it is a context-dependent effect rather than a dominant characteristic of antibody binding. The physiological significance of this phenomenon remains unclear, particularly given that Ab25 still provides robust protection in mouse models (26, 82), although these lack some human-specific factors. Overall, these findings highlight the need to consider indirect, M-protein–mediated effects when evaluating therapeutic mAbs or designing M-protein–based vaccines, while recognizing that such effects do not necessarily predict *in vivo* performance.

## Rethinking how we measure protection

### Limitations of the opsonophagocytic killing assay

Although numerous studies have advanced our understanding of host–pathogen interactions during Group A Streptococcus (GAS) infection, assessing whether vaccines or monoclonal antibodies truly confer protection remains challenging. This difficulty stems in part from reliance on surrogate assays that integrate multiple immune mechanisms into single outcome measures, thereby obscuring the specific contributions of individual effector functions.

**Figure 3:**
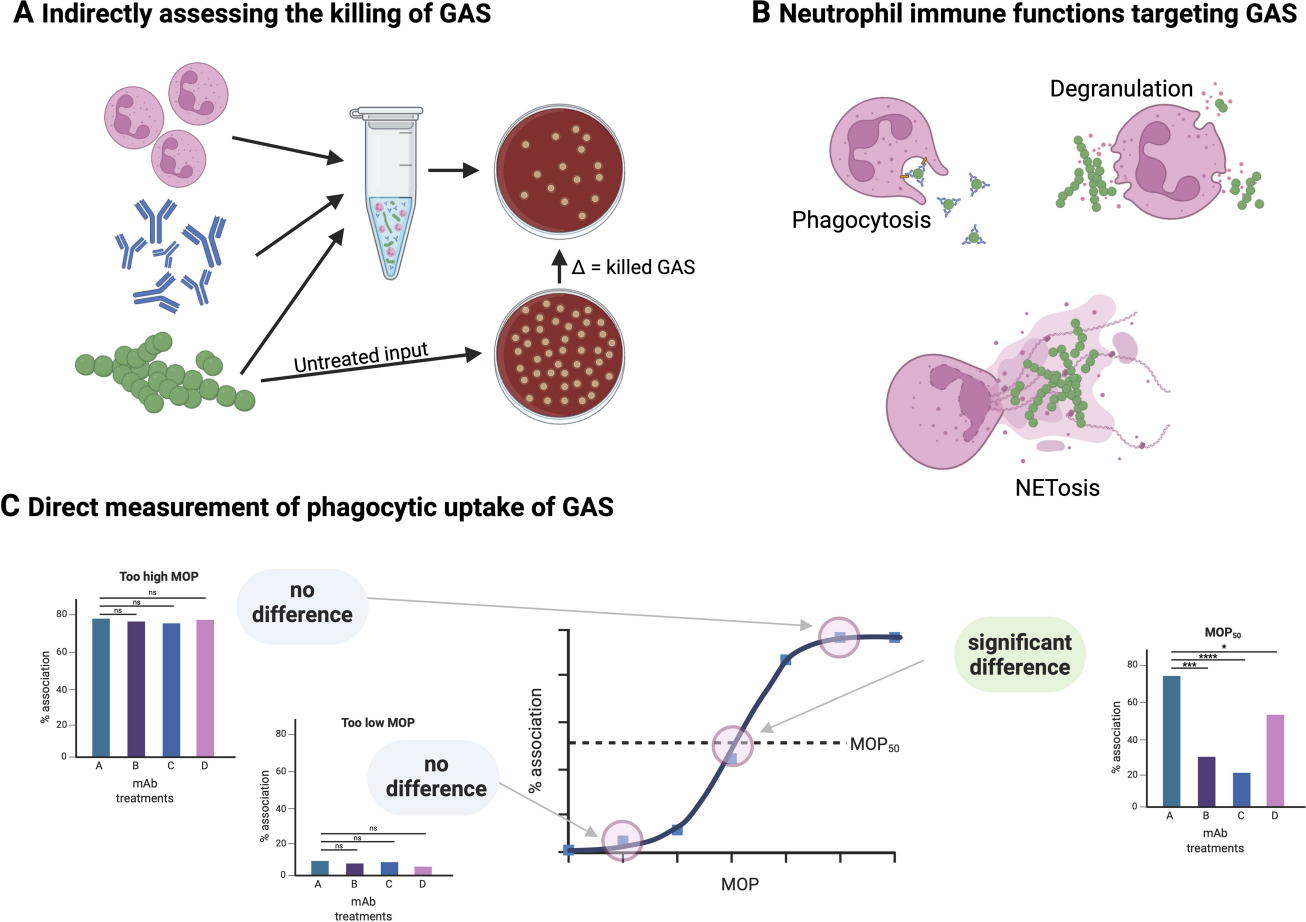
Measurement of antibody-mediated mechanisms against GAS. **(A)** The indirect bactericidal assay, commonly termed the OPKA assay, measures antibody-dependent killing of GAS by combining bacteria with a neutrophil-like cell line, antibody, and complement. After incubation, surviving bacteria are quantified by plating serial dilutions on blood agar and comparing colony counts with untreated controls. Although widely used, the OPKA integrates multiple effector pathways and provides only an endpoint readout, making it difficult to dissect specific antibody functions. **(B)** Neutrophils deploy several bactericidal mechanisms against GAS, including phagocytic uptake, granule-derived antimicrobial molecules, reactive oxygen species generation, and formation of neutrophil extracellular traps (NETs). GAS express multiple factors that interfere with these defenses, which underscores the importance of using assays capable of distinguishing opsonization efficiency from downstream killing capacity. **(C)** Quantifying the association of GAS (“prey”) with phagocytes across different multiplicities of prey (MOP) allows phagocytosis assays to be tuned for sensitivity and reproducibility. Nonlinear regression of these association curves yields the MOP50, the MOP at which 50% of phagocytes are associated with bacteria, which provides a standardized metric for comparing antibody performance across experiments, strains, or platforms (e.g., flow cytometry or microscopy). Incorporating MOP50-guided assay conditions enhances the ability to detect functional differences between monoclonal antibodies. Figure created with BioRender.com.

The opsonophagocytic killing assay (OPKA) is widely used to assess vaccine-induced “protective immunity” (83) (**Figure 3A**). In its most common implementation, GAS is incubated with neutrophil-like cells, such as differentiated HL-60 cells (84), together with complement and antibody, and bacterial survival is inferred from CFU reduction after incubation. However, despite its name, OPKA does not specifically measure killing mediated by opsonophagocytosis. Neutrophils deploy a range of antimicrobial mechanisms, including phagocytic uptake, oxidative burst, degranulation, and formation of neutrophil extracellular traps (NETs) (85) (**Figure 3B**), all of which contribute to bacterial killing in ways that cannot be disentangled by a simple CFU-based endpoint. As such, OPKA provides a black-box readout in which killing can be observed, but the underlying mechanism remains unresolved.

This limitation has led to interpretive challenges. For example, vaccine-induced antibody responses have been described as non-opsonic solely on the basis of a lack of enhanced killing in OPKA, despite the absence of any direct measurement of bacterial uptake (86). This distinction is particularly important for GAS, which—like many bacterial pathogens—possesses mechanisms that allow survival after phagocytic uptake (87, 88), although the frequency and *in vivo* relevance of intracellular persistence in human disease remain incompletely defined. Consequently, successful opsonization may not necessarily translate into bacterial killing. Additional variability arises from the tendency of GAS to aggregate, which complicates accurate CFU enumeration and further limits assay reproducibility. For these reasons, OPKA has more accurately been described as an indirect bactericidal assay (89), as it measures bacterial killing as an outcome indirectly influenced by antibodies rather than direct opsonophagocytic activity.

It should also be emphasized that OPKA is often used interchangeably with the broader concept of opsonophagocytosis assays (OPA), which encompasses a diverse range of experimental approaches. Microscopy- and flow cytometry–based assays can directly quantify bacterial association or uptake by phagocytes and, in some cases, simultaneously assess related parameters such as oxidative burst or intracellular trafficking (84, 90). Collectively, these considerations highlight the need for standardized, mechanistically resolved assays that can disentangle individual correlates of protection, including phagocytic uptake, complement deposition, toxin neutralization, and NET formation (91, 92).

### The need for standardized direct measurement of phagocytosis

Phagocytosis is a fundamental component of immune defense and tissue homeostasis, yet in the context of bacterial infection it does not necessarily equate to microbial killing (93, 94). Despite its central role, there is currently no widely accepted assay or quantitative metric for phagocytosis analogous to surface plasmon resonance–derived equilibrium dissociation constants (K_D_) for antibody binding or plaque reduction neutralization titers (PRNT_50_) for antiviral antibodies (95). As a result, phagocytosis is frequently measured but rarely standardized, limiting both reproducibility and comparability across studies, including within the GAS field.

To address this gap, we have proposed Persistent Association-Based Normalization (PAN) as a generalizable framework for quantifying phagocyte–bacterium interactions (96). PAN adapts the pharmacological concept of half-maximal effective concentration (EC_50_) to phagocytosis, but rather than measuring a terminal outcome such as bacterial killing, it focuses on an intermediate, directly observable parameter: the association between phagocytes and bacteria. In practice, bacteria are fluorescently labeled and incubated with phagocytes under defined conditions, and the fraction of phagocytes associated with bacteria is quantified by microscopy or flow cytometry.

A key feature of PAN is the systematic variation of the multiplicity of prey (MOP), the ratio of bacteria to phagocytes, while keeping experimental parameters such as opsonin concentration, incubation time, temperature, and reaction volume constant (**Figure 3C**). This approach explicitly accounts for the stochastic nature of phagocyte–bacterium encounters, often overlooked in conventional phagocytosis assays. Nonlinear regression of association data across MOPs allows determination of the MOP required to achieve 50% phagocyte–bacterium association (MOP_50_), providing a quantitative and comparable metric of opsonic efficiency.

Defining experimental conditions at MOP_50_ substantially reduces inter-assay variability and yields data that are both more reproducible and more physiologically informative. Importantly, PAN does not replace downstream functional assays but instead provides a standardized framework for them. Parameters such as internalization, intracellular survival, complement deposition, or killing can be assessed under MOP_50_-defined conditions, enabling clearer interpretation of antibody-mediated effects. Moreover, PAN is assay-agnostic and can be integrated with microscopy-based analyses, flow cytometry, or indirect bactericidal assays. In this way, PAN offers a flexible yet rigorous approach to standardizing phagocytosis measurements and improving the interpretability of antibody function in GAS immunobiology.

### Animal models and their limitations in GAS research

An important step in developing antibody therapies is testing in animal models. However, GAS is a strict human pathogen and fails to cause its typical diseases in other species, making this step challenging. Murine infection models are commonly used but can be hard to establish. Disease often requires highly virulent or adapted GAS strains delivered at unrealistically high inocula to mice, which poorly reflects natural human infection (97). Moreover, the route of inoculation in mice can skew GAS virulence factor expression (including factors targeting antibodies), so an antibody’s efficacy might depend on the model’s particulars (98). Despite these limitations, conventional murine systems, including subcutaneous and systemic challenge models, have provided useful insights. For example, the human mAb Ab25 previously mentioned reduced bacterial burden and improved survival in infected mice in two different models (26, 82).

However, standard mouse models may underestimate the potency of human IgG antibodies, because mouse Fcγ receptors and complement interact differently with human IgG, and because GAS-secreted enzymes like EndoS and IdeS can inactivate antibodies *in vivo* (98). To address these issues, transgenic humanized plasminogen mice, which express human plasminogen in place of the murine form, have revealed enhanced bacterial dissemination and recapitulate the pathogen’s dependence on human plasminogen for invasive disease (99). Similarly, humanized immune models expressing human FcγRs, CD46, or HLA molecules better reproduce human antibody effector functions; notably, Pandey et al. demonstrated that dual anti-M (J8 peptide) and anti-SpeC antibodies could resolve established streptococcal toxic shock in HLA-humanized mice (81). Meanwhile, non-human primate models (macaque pharyngitis) remain the most physiologically relevant for GAS throat infections, since macaques develop tonsillitis and immune responses mirroring human infection (100). Each model yields useful insights but also underscores the need to further refine our *in vivo* experimental systems to faithfully mimic human-specific host–pathogen dynamics.

The serious lack of human-translatable experimental findings has led to a renewed debate about human infection models. In fact, the first landmark GAS human pharyngitis study was recently completed in 2019 (101, 102). However, even if it is now possible to carry out trials that are safe enough to reliably exclude invasive infections and postinfectious autoimmune sequelae among participants, such experiments would still be highly complex and costly, limiting access to only a small part of the GAS research community. Therefore, developing more advanced *in vitro* and *ex vivo* human infection models is crucial to address this gap. In this context, human tonsillar epithelial and immune organoid models have emerged as promising human-relevant systems that recapitulate key features of tonsillar tissue architecture, epithelial barriers, and local immune responses at the primary site of GAS colonization (103). Although their application to GAS is still in its infancy, studies conducted with other human pathogens have shown that these models offer considerable potential to study GAS pathobiology and host–pathogen interactions, as well as to serve as translational platforms for immunobiological studies and therapeutic or vaccine screening (104).

### Monoclonal antibodies as a treatment option

#### Designing effective mAb therapies for GAS

Modern monoclonal antibody discovery has been transformed by single B-cell screening technologies, which preserve native heavy- and light-chain pairing and enable rapid identification of physiologically relevant human antibodies (105). When coupled with advanced bioreactors and high-density culture systems, these methods now enable scalable, cost-effective production of clinical-grade mAbs (106), accelerating translation from discovery to therapy.

Despite promising preclinical results targeting GAS virulence factors such as streptolysin O (SLO) (107), fibronectin-binding proteins (e.g., Fba (77)), and superantigens (polyclonal (81)), no monoclonals have advanced to clinic use. This is likely due to a combination of factors: (i) the complexity and heterogeneity of GAS infections; (ii) difficulty in identifying appropriate patient populations, many of whom present in late-stage sepsis; and (iii) the narrow scope of some targets, which fail to account for the multifactorial nature of GAS pathogenesis. These challenges highlight the need for antibodies that not only neutralize toxins or enzymes but also promote robust bacterial clearance.

**Figure 4:**
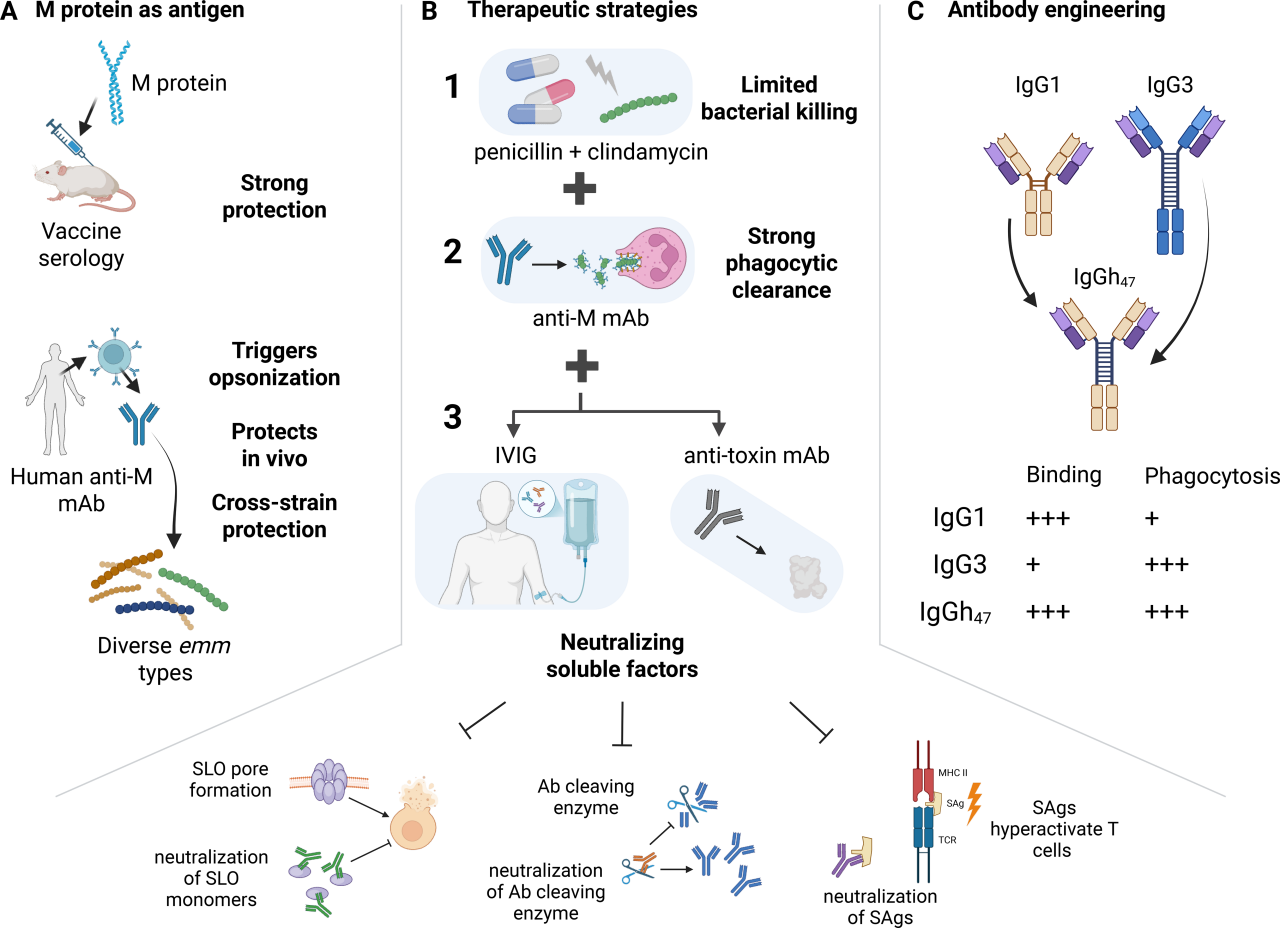
M protein as a primary target for therapeutic antibody development. **(A)** Rationale for prioritizing monoclonal antibodies against M protein. In both human serology and vaccine challenge models, antibodies against M protein consistently serve as the *positive control* for opsonic and protective activity, whereas antibodies against alternative GAS antigens, such as SLO, SpyCEP, IdeS, or superantigens, show more variable or limited efficacy. Recent isolation of human anti–M protein monoclonal antibodies has demonstrated broad opsonization across multiple *emm* types and protection in mouse models, providing strong preclinical support. These data suggest that M protein–directed antibodies represent the current most reliable foundation for a therapeutic approach. **(B)** Combination therapeutic strategies. Despite widespread use, intravenous immunoglobulin (IVIG) has not consistently improved outcomes in invasive GAS disease, in part because polyclonal IgG preparations lack sufficient titers of opsonic anti–M protein antibodies and fail to mediate robust bacterial killing. Nonetheless, IVIG may contribute clinically by neutralizing soluble virulence factors such as toxins, superantigens, and shed M protein–fibrinogen complexes. An optimized therapeutic strategy may therefore combine a potent anti–M protein monoclonal antibody, with high opsonophagocytic capacity, with either (i) standard-of-care antibiotics alone (1 + 2), or (ii) antibiotics plus adjunctive IVIG or mAbs targeting specific factors (1 + 2 + 3) to simultaneously neutralize soluble factors while the anti-M antibody clears the bacteria. **(C)** Engineering anti–M protein antibodies for enhanced effector function. Fc- and hinge-region engineering can substantially increase the ability of anti–M protein antibodies to promote phagocytosis and bacterial killing. Variants incorporating IgG3-derived hinge elements or extended hinge designs (e.g., IgGh47) enhance flexibility and Fc receptor engagement, leading to markedly improved opsonophagocytic killing *in vitro*. Such engineered backbones may be especially valuable for generating next-generation anti–M protein therapeutics with superior potency and breadth. Figure created with BioRender.com.

In this context, the M protein stands out as a uniquely suited target (**Figure 4A**). It is abundantly expressed on the GAS surface and plays a central role in immune evasion, host protein binding, and strain-specific as well as conserved immune responses. The human mAb Ab25 has demonstrated both cross-*emm*-type reactivity and potent phagocytosis enhancement (26), addressing two key clinical requirements: functional clearance and breadth of protection. Furthermore, M protein is a validated immunogen in multiple successful vaccine studies (108, 109), reinforcing its therapeutic relevance.

Although a synthetic monoclonal antibody (mAb) cocktail that fully mimics the polyclonal immune response would be ideal, manufacturing, regulatory, and cost constraints currently limit the feasibility of such approaches. Even oligoclonal or bispecific formats, while conceptually attractive, introduce additional complexity in formulation and clinical approval. As a result, a strong case can be made for prioritizing a single, broadly opsonic anti–M protein monoclonal antibody as the backbone of a therapeutic strategy, administered in combination with antibiotics. This core therapy could, in the future, be augmented with adjunctive mAbs targeting key virulence factors such as streptolysin O (SLO), IdeS/EndoS, or superantigens in severe disease manifestations like streptococcal toxic shock syndrome (**Figure 4B**). Such combination strategies are further supported by the potential synergy between therapeutic antibodies and conventional antibiotics. Antibody-mediated remodeling of the bacterial surface could enhance antibiotic penetration, while accelerated immune clearance may reduce bacterial burden to levels that limit both disease severity and the emergence of resistant variants. There is also clinical precedent, as mAbs have been deployed as adjuncts rather than replacements for antibiotics. For example, in inhalational anthrax, the FDA-approved monoclonal antibody raxibacumab is indicated in combination with antibiotics (110), as antibiotics alone cannot neutralize toxin-mediated pathology (111).

At the same time, we acknowledge that the extensive genetic and antigenic diversity of GAS presents an inherent challenge for monoclonal therapies. A single epitope-specific mAb, regardless of its breadth, may not cover all circulating strain variants, and selective pressure could promote immune escape. While large antibody cocktails remain difficult to implement, smaller combinations of complementary mAbs or bispecific antibodies represent a pragmatic intermediate solution, offering broader coverage while mitigating resistance risk.

An advantage of combining antibodies extends beyond expanding epitope coverage to include functional synergy between distinct antibody specificities. In polyclonal settings, antibodies targeting different epitopes can cooperate to enhance effector mechanisms such as complement activation or opsonophagocytosis. A well-established example is the anti–Neisseria meningitidis factor H–binding protein monoclonal antibodies JAR3 and JAR4, which are individually non-bactericidal yet mediate potent complement-dependent killing when combined (112). These findings illustrate that antibody combinations can yield emergent protective functions not predictable from the activity of individual clones alone. Notably, even a single therapeutic mAb may benefit from such synergy by cooperating with naturally occurring or infection- or vaccine-induced antibodies present in the host. Together, these points support the rationale for both carefully designed mAb combinations and the deployment of broadly opsonic monoclonals that can integrate into and amplify existing humoral immune responses.

Importantly, this targeted approach contrasts with the limited and inconsistent efficacy observed for intravenous immunoglobulin (IVIG) in invasive GAS disease (42), which likely reflects variability in donor antibody repertoires and insufficient titers of opsonic anti–M protein or anti-superantigen antibodies (113). In contrast, a rationally selected and engineered anti–M protein monoclonal antibody offers defined specificity, reproducible potency, and the potential for optimized effector function, thereby providing a more predictable safety and efficacy profile than polyclonal preparations.

#### Engineering mAbs for enhanced therapeutic potential

In the past decades, mAb therapies have become increasingly popular therapeutic agents. There are currently over 200 approved mAbs, with many more in clinical trials (114). An important consideration when developing a mAb therapy is the choice of Ab subclass, since this can have vast implications for its immune function. Of these, the vast majority are produced as the subclasses IgG1 and IgG4. This is mainly due to their favorable immune profile in relation to cancer therapy, where it is important for a mAb to efficiently trigger antibody-dependent cellular cytotoxicity (ADCC) (115). However, in a recent study, we examined class-engineered variants of Ab25 (originally IgG1) by expressing it as all four human IgG subclasses. Strikingly, the IgG3 version of Ab25, despite exhibiting slightly lower antigen binding affinity, mediated vastly higher opsonophagocytic killing of GAS (manyfold increase) (**Figure 4C**). This was attributed to IgG3’s elongated, flexible hinge region. By incorporating the extended IgG3 hinge region into the IgG1 version of Ab25 (creating hybrid IgGh47), we obtained a mAb that combined IgG1’s high affinity with IgG3’s enhanced opsonic function, leading to superior protective efficacy in the mouse model (82). This exemplifies the potential of antibody engineering to optimize anti-GAS mAbs. Others have reported that it was possible to improve IgG3 complement activation against meningococci by shortening the hinge region to that of IgG1 (116), indicating that antibody effector functions are likely to be both context- and epitope-dependent. Insights gained from decades of mAb development in cancer and viral therapeutics can likely be leveraged to further design highly efficacious and safe GAS-targeted mAb therapies.

### Combating the global threat of GAS: a multifaceted approach that includes monoclonals

Developing effective therapies for GAS has become an urgent global health priority. The increasing disease burden, emerging antibiotic resistance, and severity of invasive GAS infections all highlight the vulnerability of current approaches. Penicillin remains surprisingly effective as a first-line treatment, but reports of GAS with reduced β-lactam sensitivity and rising clindamycin resistance warn that even formerly ‘uncomplicated’ infections may become challenging to treat. The development of an effective vaccine, combined with monoclonal antibodies, could significantly reduce the impact of GAS, both in prevention and acute therapy. Advances in antibody engineering (e.g. fully human mAbs and Fc engineering) and *in silico* modeling open new avenues for highly targeted therapies. Simultaneously, progress will require interdisciplinary efforts – from improved assay standardization to better human infection models – to accurately evaluate efficacy. Ultimately, prioritizing GAS-focused vaccine development and novel immunotherapies, while ensuring equitable global access, will be crucial to mitigating the GAS disease burden in vulnerable populations. By advancing a coordinated, multidisciplinary research agenda, we can revolutionize the clinical management of GAS infections and improve patient outcomes globally.
